# Individuals’ Perceptions as a Substitute for Guidelines and Evidence: Interview Study Among Clinicians on How They Choose Between In-Person and Remote Consultation

**DOI:** 10.2196/35950

**Published:** 2022-05-25

**Authors:** Amia Enam, Heidi C Dreyer, Luitzen De Boer

**Affiliations:** 1 Department of Industrial Economics and Technology Management Faculty of Economics and Management Norwegian University of Science and Technology Trondheim Norway

**Keywords:** video consultation, work routine, outpatient care, telemedicine, clinician, professional work

## Abstract

**Background:**

Video consultation (VC) is increasingly seen as a cost-effective way of providing outpatient care in the face of dwindling resources and growing demand for health care worldwide. Therefore, the sustainable implementation of VC is a phenomenon of interest to medical practitioners, researchers, and citizens alike. Studies are often criticized for not being sufficiently robust because the research settings are mostly small-scale pilot projects and are unable to reflect long-term implementation. The COVID-19 pandemic has compelled clinicians worldwide to conduct remote consultation, creating a favorable context to study large-scale remote consultation implementation.

**Objective:**

The aim of this study was to thoroughly investigate how clinicians reason their choice of different consultation modes in the routine of consultation and what the underlying reasons are for their choices. We posited that a deeper understanding of clinicians’ perceptions of remote consultation is essential to deduce whether and how remote consultation will be adopted on a large scale and sustained as a regular service.

**Methods:**

A qualitative approach was taken, in which the unit of analysis was clinicians in one of the largest university hospitals in Norway. In total, 29 interviews were conducted and transcribed, which were used as the primary data source. Using the performative model of routine as the theoretical framework, data were analyzed using deductive content analysis.

**Results:**

Clinicians have mixed opinions on the merits and demerits of VC and its position between in-person and telephone consultation. Totally, 6 different planning criteria were identified, and individual clinicians used different combinations of these criteria when choosing a mode of consultation. The ideals that clinicians hold for conducting consultation can be divided into three aspects: clinical, interpersonal, and managerial. VC engenders a new ideal and endangers the existing ideals. VC causes minor changes in the tasks the clinicians perform during a consultation; thus, these changes do not play a significant role in their choice of consultation. Clinicians could not identify any changes in the outcome of consultation as a result of incorporating a remote mode of consultation.

**Conclusions:**

Clinicians feel that there is a lack of scientific evidence on the long-term effect of remote consultation on clinical efficacy and interpersonal and managerial aspects, which are crucial for consultation service. The absence of sufficient scientific evidence and a clear understanding of the merits and demerits of VC and standard practices and shared norms among clinicians regarding the use of video for consultation both create a void in the consultation practice. This void leads clinicians to use their personal judgments and preferences to justify their choices regarding the consultation mode. Thus, diverse opinions emerge, including some paradoxical ones, resulting in an uncertain future for sustainable large-scale implementation, which can reduce the quality of consultation service.

## Introduction

### Background

Video consultation (VC) is increasingly seen as a cost-effective way of providing outpatient care in the face of dwindling resources and growing demand for health care worldwide [1,2]. Several pilot studies have reported VC to be beneficial while providing health care access to patients in rural areas with insufficient care providers [3,4], thus making the consultation time-efficient [5], reducing the need for travel for patients [6,7], and providing the ability to add care providers from different locations and family members as needed to provide coordinated care [4]. Therefore, the sustainable implementation and adoption of VC is a phenomenon of interest to medical practitioners, academic researchers, and citizens alike. Studies on VC have taken several trajectories, such as measuring efficacy, diagnosis-specific outcomes, and safety. However, these studies are often criticized for not being sufficiently robust because the research settings are often small-scale pilot projects or interventions and, therefore, are unable to reflect long-term implementation [2,8]. To address this gap, we focused on a hospital where VC is no longer a trial project but is gradually becoming a regular service. We aimed to understand how, in their regular work routine, clinicians choose a particular mode of consultation when three alternative modes—in person, video, and telephone—are available to conduct a consultation. Clinicians are the ultimate decision makers in adopting or abandoning technology in hospitals [9]; therefore, they are the focus of this study. The pandemic has compelled clinicians worldwide to use remote consultations through telephone and video. Therefore, clinicians have gained substantial experience in conducting remote consultations. Thus, the pandemic has created a favorable context to study how clinicians choose the consultation mode. In contrast, as pandemic restrictions are being lifted, it is crucial to investigate how clinicians are making sense of the situation and how this may impact remote consultation implementation. Henceforth, we have used the term *remote consultation* to imply both video and telephone consultation and the abbreviations *PC*, *VC*, and *TC* to imply in-person, video, and telephone consultation, respectively.

### Previous Studies

Although there is a lack of in-depth studies considering the intricacies of remote consultation implementation, recent studies have focused on the process of implementation. The nonadoption, abandonment, scale-up, spread, and sustainability framework aims to assist and evaluate the success of technology-enabled health care programs through pragmatic questions focusing on seven domains: condition or illness, technology, value proposition, adopter system, organizations, wider (institutional and societal) context, and interaction and mutual adaptation among all these domains over time [10]. An extension of this framework—the planning and evaluating remote consultation services method—has been developed for VC. This framework evaluates the following domains: reason for consulting, patient, clinical relationship, home and family, technologies, staff, health care organization, and wider system [11]. These frameworks offer a comprehensive method for planning and evaluating implementation. However, the mechanisms that drive or limit the implementation process are not the focus of these frameworks.

Nonetheless, studies on how VCs have expanded during the pandemic has discussed these mechanisms, positing that the reasons for successful expansion include the national-level groundwork conducted before the pandemic, a strong strategic vision, a well-resourced quality improvement model, dependable technology, and multiple opportunities for staff to try the video option [8]. However, these results are only from the pandemic period. As this is a special situation and does not reflect normal conditions, it does not shed light on the future of VC when the pandemic no longer limits citizens’ movements. A prepandemic study by Greenhalgh et al [12] investigated the real-world implementation of VCs and concluded that (1) although clinicians consider VC to be safe, effective, and convenient for some patients in certain situations, those situations are rare compared with the overall number of outpatient consultations and (2) it is challenging to embed VC into the routine practice of consultation when clinicians are hesitant to change.

A recent literature review indicated that empirical studies focusing on VC implementation did not identify the distinct processes essential for achieving large-scale adoption of VC [13]. We argue that how clinicians choose different modes of consultation is an essential process in the long-term adoption of VC. Clinicians are empowered with expert knowledge that is inaccessible to people outside the clinical profession; thus, clinicians decide both the definition of the goals (eg, what is quality of care) and the means to reach the goals (eg, how the quality of care can be attained) [14]. On the one hand, clinicians have codified knowledge and standard practices based on scientific evidence. However, on the other hand, they have shared values and norms that are seemingly flexible, yet uniformly shared and strongly held [15]. Therefore, clinicians play a crucial role in the implementation of any technology in hospitals, and their role may even be more significant for VC adoption because, mostly, a consultation is a one-to-one service between the clinician and patient. We posit that a deeper understanding of clinicians’ perceptions of remote consultation is essential to deduce whether and how VC will be adopted on a large scale and sustained as a regular service. Hence, this study focused on how clinicians decide on the mode of consultation in their regular work routines. We used the performative model of routine as the theoretical framework, as explained in the following section.

### Theoretical Framework: Performative Model of Routines

**Figure 1:**
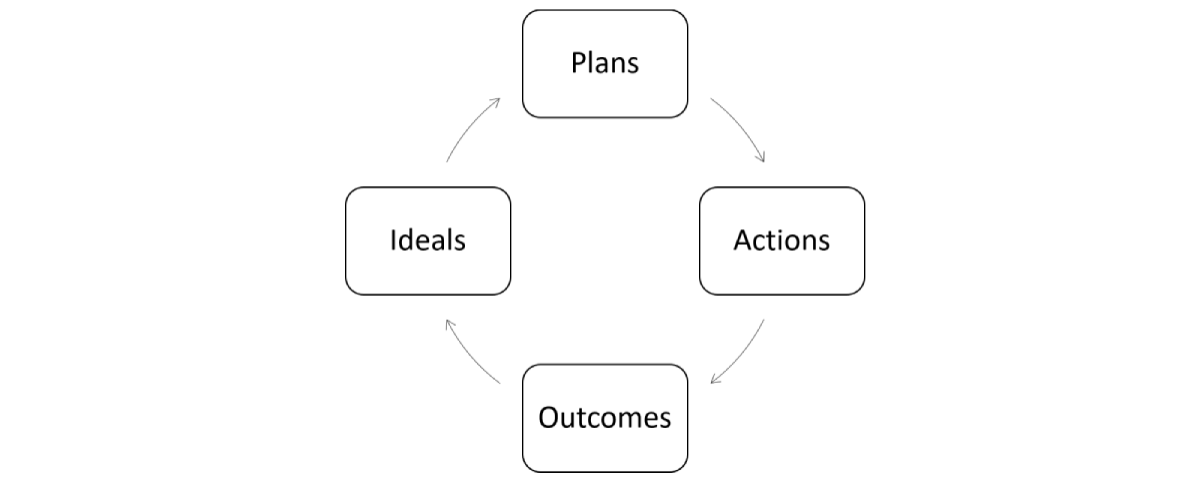
Performative model of routine [21].

Studying information technology (IT) implementation from a professional’s routine perspective can provide a better understanding of the long-term applications of IT [16]. The tasks within an organization are accomplished through temporal structures known as routines [17], standard operating procedures [18], and habits [19]. “An organizational routine is not a single pattern but, rather, a set of possible patterns—enabled and constrained by a variety of organizational, social, physical and cognitive structures—from which organizational members enact particular performances” [20]. Routines work as both stabilizing force and apparatus to evolve with changing environmental demands in an organization [16]. We used the performative model of routine [21] to identify how the implementation of VC impacts the routine of consultation. Figure 1 shows how the equilibrium of the state of routines can change and follow a new pattern in organizations; this model has been used in different studies as a theoretical lens for studying human-technology interaction [22], along with how people make sense of the changing organizational goals [23].

According to the performative model of routine, routines are not fixed actions performed by the people in an organization; rather, they are dynamic patterns stemming from ongoing exchanges between ideals, plans, actions, and outcomes. Ideals represent normative influences including values, goals, missions, and expectations. Plans are thoughts and intentions that cause the actions. Plans and actions generate the outcome. The outcomes are then compared with the ideals to set the next course of plans and actions. None of these 4 aspects are immune to change. Even ideals can be altered if the generated outcomes—whether intended or unintended—reveal new possibilities. The people in an organization change the routine when they see that the outcomes of the ongoing routines are either falling short of the ideals or showing the possibility of new ideals. When the outcomes fall short of the existing ideals, the actors strive to change their plans and actions to attain the ideals. When the outcomes show the possibilities of new ideals, the actors expand their plans and actions to fulfill the new ideals. We used this model to frame how clinicians make sense of the implementation and continuation of the large-scale application of VC, along with TC and PC, in hospitals.

This study aimed to thoroughly investigate how clinicians reason their choice of different consultation modes, namely, PC, TC, and VC. The performative model of routine provided us with a systematic structure to analyze clinicians’ choices regarding different consultation modes. On the basis of the findings, this paper also explained the underlying reasons for the clinicians’ choice. The following was the guiding research question: How do clinicians choose the consultation type from among PCs, VCs, and TCs?

## Methods

### Overview

A qualitative approach was taken, in which the unit of analysis was the clinicians in one of the largest university hospitals in Norway, which is anonymized as the Pioneer Hospital in this paper. We purposefully chose this hospital because it provides a rich ground to investigate our research question. As the name suggests, the hospital is a pioneer in promoting and deploying cutting-edge digital tools to provide and manage health care services in the region. An active research and development center works closely with the hospital management to maintain a progressive approach regarding the use of technologies in health care, and the hospital has a substantial financial budget for the innovation and implementation of new technologies. As the Pioneer Hospital has a long tradition of innovating and designing IT-enabled services, it can be presumed that there are fewer managerial and organizational challenges to the implementation of VC when compared with a hospital that has little or no experience with the implementation of new technology. A hospital that seemingly has few managerial and economic challenges in implementing VC can provide us with the opportunity to look beyond the financial and organizational challenges mentioned in previous studies [12] and focus on how clinicians choose between different modes of consultation. In the following sections, we have discussed the context of the study and explained the data collection and analysis process.

### Research Setting

Amid resource constraints and an increasing demand for health care, the health authority in Norway has decided to implement VC as an alternative to PC in hospitals countrywide. The goals of implementing VC are to (1) reduce the cost incurred from a patient traveling to and from the hospital for PC and (2) reduce the travel-induced stress and other activities that a patient may need to consider (eg, taking leave from work and managing childcare) [24]. Following the mandate of the health authority, the Pioneer Hospital started to prepare to implement VC and decided to use Skype for Business (Microsoft Corporation). This was a strategic decision because this software had been in use in the Pioneer Hospital previously for long-distance meetings. Therefore, the people working in the hospital, including clinicians, were familiar with the technology and video calling options. After buying adequate accessories (eg, headphones and video cameras), the hospital started to implement VC in 2019. The clinicians were not involved in planning or designing the VC implementation. They were also not forced to adopt VC. Initially, the advisers reached 3 of the department heads to ask their clinicians to conduct VC as a pilot project. The plan for the pilot project was made by the advisers and department heads, who were also clinicians and conducted consultations. The clinicians were given the freedom to decide whether and when VC is suitable. No particular goal (eg, minimum number of VCs) or time frame was given for the project. Before the introduction of VC, the hospital had two modes of consultation: PC and TC. However, TC was used as an impromptu way of contacting the patient, specifically when a quick call to the patient seemed to be more practical than waiting for weeks—or even months—for a scheduled consultation. The hospital was not paid for these TCs, and no records of the number of TCs made by the outpatient clinics were maintained.

At the beginning of 2020, the hospital asked all the departments to start conducting VCs. Similar to the pilot project, the clinicians were given the freedom to decide on the mode of consultation. However, this time, an annual goal was set for the departments, not for the individual clinician. As mentioned by both advisers and clinicians, there was no penalty or consequence for not being able to meet the goal. Currently, a plan is made to provide additional budgets to the departments that meet the goal in the future. At the beginning of VC implementation, the health authority changed the reimbursement plan for how the clinics were paid (by the government) for the consultations. According to the new plan, VC and TC were reimbursed with 75% and 67% of that received for PC, respectively. Despite the request to use VC and this change in the reimbursement plan, the clinicians were sluggish in using VCs until the COVID-19 pandemic hit the country and a nationwide lockdown was announced in March 2020. The restraint on movement stopped the patients from visiting the clinics for consultations, and the hospital was only allowed to admit patients with emergency issues. Therefore, clinicians were compelled to conduct VCs more frequently than before the lockdown. As the lockdown continued, the health authority revised the reimbursement plan again, this time, providing equal pay for all modes of consultation. This plan has remained active so far, irrespective of the changes in the strictness of the lockdown. Textbox 1 provides an overview of the time line of VC implementation in the hospital since 2019.

**Implementation of video consultation (VC) at the Pioneer Hospital (2019-2021).**

**2019**

- Identified VC to be a solution (1) for reducing the cost of public health care service by reducing the need for patients to travel to hospital for consultations and (2) by reducing travel-induced stress and other activities for patients.
- Planned and arranged the necessary software and hardware for VC.
- Conducted pilot projects for VC.

**2020 (before the pandemic and lockdown)**

- Set a goal for the number of VCs for each department after discussion with the department heads.
- Started to receive incentives for telephone consultation and VC, at rates of 67% and 75% of an in-person consultation, respectively.

**2020 (from the beginning of the pandemic and lockdown)**

- Changed the incentive to be equal for all modes of consultation.
- Revised and scaled up the goals for VC.
- Total number of VCs increased from 200 (in 2019) to 2000.

**Present (October 2021)**

- Planned to give an additional budget to the departments that reach the annual target of VC by the end of the year.
- Planning to establish VC as a regular alternative to in-person consultation in standard patient pathways.
- Saved NOK 52,000,000 (US $5,362,318) in traveling costs.

### Ethics Approval

This study was approved by the Norwegian Center for Research Data (NSD; reference number 800636). The committee assessed the application and decided that “the processing of personal data in this project will comply with data protection legislation, presupposing that it is carried out in accordance with the information given in the Notification Form and attachments, dated 08.10.2019. Everything is in place for the processing to begin.” We also sought permission (reference number 58059) from the Regional Committee for Medical and Health Research Ethics (REK) for interviewing patients. However, the committee assessed that the project falls outside the scope of the Health Research Act (ACT 2008-06-20 number 44); thus, it could be conducted without the REK’s approval. Following the NSD guidelines, written consent was obtained for each interview, and data were anonymized and stored on the researcher’s server at the university where the project was conducted.

### Data Collection and Analysis

A semistructured interview technique was used to collect data. Following the checklist provided by the consolidated criteria for reporting qualitative research [25] and the case study protocol guidelines provided by Yin [26], we developed an interview guide (Multimedia Appendix 1). The guide includes three sets of questions to be asked to the clinicians, patients, and advisers, respectively. The interview guide aimed to include all questions that could capture the complexities and dynamic character of the clinicians’ routines of consultation and VC implementation process. The questions were open ended, and the focus was to gather information on (1) the VC implementation process, (2) how the implementation of VC changes the consultation process, and (3) the perceptions of VC. To create a broad array of questions, we did not follow any particular framework at this stage, but instead, outlined the questions following different studies, including the performative model of routine [21], technology acceptance models [27,28], and structurational model of technology [29]. The interview guide was submitted to the hospital authority, NSD, and REK before data collection began. The questions were approved without changes. However, we added question number 8 for the clinicians after the first interview because that interview revealed that the duration of consultation may vary and that the documentation of patient records requires substantial amount of time.

We studied VC implementation in the Pioneer Hospital since the fall of 2019 and interviewed a group of clinicians (n=16), advisers of the research and development center responsible for facilitating VC implementation (n=7), and patients (n=16). We selected these 3 groups because they have the best knowledge of implementing, conducting, and receiving VC service, which is an essential criterion for selecting the sample [30]. All the advisers involved in VC implementation in the hospital were interviewed. To recruit clinicians and patients, we sent invitation letters to them, asking them to participate. The inclusion criterion was that they had experienced at least one mode of remote consultation, that is, TC or VC, at least once in the past 6 months. The hospital’s communication channel was used to send invitation letters via email. To determine the number of clinicians and patients in the respective sample group, we relied on data saturation—the point of time when information from the informants becomes repetitive and no further information can be gained from further data collection [30]. Therefore, to recruit enough informants, the invitation letter was sent twice to clinicians and thrice to patients, leaving an interval of 2 months in between. The interviews were a mix of face-to-face and video calls, following the pandemic guidelines in the region. The face-to-face interviews were audio-recorded, and video calls were video-recorded. Documents and nonparticipatory observation methods [31] were used to gather contextual information. A wide range of reports on the digitalization of the hospital, published between August 2019 and August 2021, was scrutinized. These reports can be divided into two categories: (1) public reports published by the government and (2) internal reports published by the hospital. The first group of reports provides the macrocontext of VC implementation, presenting how the government is planning and strategizing different digital health services, including VC [24,32,33]. The second group of reports provides the ongoing status of VC implementation in the hospital, including the numbers of PC, VC, and TC, along with the future goals for these consultation modes. To maintain confidentiality, these reports are not cited. Furthermore, the first author (AE) participated and took notes in a workshop in which clinicians shared presentations of their experience of using VC with the top management. Subsequently, the author gained access to those presentations.

In this study, the primary data source was interviews with clinicians, whereas the other interviews, documents, and observation notes were used for contextual understanding and data triangulation [34]. Data triangulation was performed to enhance the quality of the data used and strengthen the findings of the study [26]. We interviewed the clinicians twice: once in the middle of the pandemic (2020) and once when the pandemic-induced restrictions were lifted in Norway (2021). Of the 16 clinicians, 3 (19%) clinicians could not participate in the second round of interviews for different reasons, resulting in a total of 29 interviews. The first round of interviews lasted between 60 and 75 minutes, and the second round lasted approximately 45 minutes. All the interviews were recorded and transcribed. We contacted 19% (3/16) of the clinician-informants via email after the transcribing process to obtain some clarification on certain issues mentioned in the interview.

To keep data analysis transparent and easy to understand, we followed the criteria from the consolidated criteria for reporting qualitative research framework, which suggests reporting on the number and roles of data analysts and the derivation of themes and performing participant checking [25]. Initially, all the recordings of the interviews were transcribed verbatim by the first author (AE) to minimize interviewer bias. The interview transcripts were then read several times to gain familiarity with the content, and a comprehensive narration of the case was written and shared with the other 2 authors (HCD and LdB). A narrative strategy is often used in qualitative studies to organize data and increase contextual understanding [35]. The remaining data analysis can be divided into two parts: (1) mapping the VC implementation process and (2) mapping the clinicians’ routines for conducting consultation services. To map the VC implementation process, we used a visual mapping strategy that is beneficial to arrange data from different sources sequentially and against the time line [35]. Therefore, we plotted the events that occurred regarding the implementation of VC. We plotted these events as narrated by our informants and as described in public and internal documents. We used pen and paper to map the process. Textbox 1 presents a schematic version of the implementation process.

For the second part of data analysis, we used the deductive content analysis method, which “...aims to test existing categories, concepts, models, theories, or hypotheses...in a new context” [36]. The performative model of routine was applied as the theoretical framework to guide our analysis. The four aspects of the model (ie, ideals, plans, actions, and outcomes) were used to color-code the quotes in the clinician’s interview, and then, those quotes were grouped under these 4 aspects in an Excel spreadsheet. This process was conducted separately for the first and second rounds of the interviews to assess whether their perceptions and routine have changed over time. During this process, the coauthors investigated different quotes obtained from the clinicians and discussed their meaning to ensure that the researchers’ personal biases were minimized. Moreover, no change in routine or perception was identified between the two rounds of interview. The notes made from the documents and experience-sharing webinar were then cross-matched with the data content in the Excel sheet. Finally, the narrations of advisers and patients were thoroughly read and compared with the clinicians’ data content to identify discrepancies among the clinicians, advisers, and patients. For example, we asked both clinicians and patients how they decided on the mode of their next consultation. Therefore, we compared the answers provided by patients with those provided by clinicians to identify any discrepancies. Similarly, we compared the advisers’ responses to whether a guideline is provided to the clinicians on when to use which mode of consultation with the response of clinicians. Subsequently, our analysis was presented to the study participants in two meetings at the hospital and two digital meetings with the advisers and clinicians, to ensure that the researchers were not misinterpreting the data or misusing the quotes. The feedback received from these meetings was considered for further refinement by changing a few words in the findings, so that they were easier to understand.

## Results

### Overview

In this section, using the performative model of routine, we identified how the clinicians chose different modes of consultation. First, we have provided a list of ideals, plans, actions, and outcomes (Textbox 2), and then, we have explained and analyzed them with exemplar quotes.

**List of ideals, plans, actions, and outcomes in the routine of consultation, as described by the clinicians.**

**Ideals**

- Right diagnosis.
- Right course of action for treatment (ie, laboratory test and medicine).
- Good communication and conversation with the patient.
- Making patients feel safe and comfortable about the diagnosis and the treatment.
- Reducing patients’ stress or need to adjust the daily schedule for traveling to the hospital.

**Plan**

- Whether physical examination is needed in the next consultation.
- Whether telephone consultation (TC) is more efficient than video consultation (VC) for this consultation because it has low need for technical ability and the consultation room does not need to be equipped with microphone, speaker, and camera.
- Whether VC is more efficient because the patient can be seen to an extent.
- Where the patient lives.
- Whether the patient will be able to use the technology for VC and understand and respond to the instructions over a video call.
- Whether making a telephone call instead of a video call can add any benefit for the clinician, for example, by taking the call from home or after clinic hours.

**Action**

- Checking the patient’s history and referral immediately before inviting the patient into the consultation room or a day before, depending on the time available to the clinician and complexity of the case.
- Bringing the patient into the room (for in-person consultation [PC]), calling the patient using a telephone (for TC), or logging in for the VC and admitting the patient from the web-based waiting room to the web-based consultation room.
- Troubleshooting technical issues both at the clinician’s and patient’s end. If the technical issue (most often at least one party cannot see or hear the other) persists, either calling the patient by phone immediately or rescheduling the consultation (for VC).
- Opening up the conversation and conducting clinical triage.
- Conducting a physical examination (only for PC).
- Taking notes on paper or computer.
- Prescribing medication and ordering tests.
- Discussing the time of the next consultation (this step is irregular).
- Filling the reimbursement form and giving it to the patient (for PC) or the health administrator at the end of the day (for VC and TC).
- Filling the details of the consultation in the patient’s electronic health record.
- Submitting the completed form to the system.

**Outcome**

- Patients are diagnosed correctly and appropriate treatment is started.
- Laboratory tests are ordered to further investigate the patient’s health status.
- The laboratory report is discussed with the patient, and suitable treatment is started.
- The effects of treatment are checked, and the course of future treatment is set.

### Clinicians’ Choice of Consultation Type: PC, VC, and TC

#### Ideals and Outcomes

Outcomes and ideals are closely related because ideals are the desired outcomes. Therefore, we have discussed these 2 aspects together. In the Pioneer Hospital, the clinicians did not feel the need for a change in consultation routine before VC was introduced. Therefore, VC was an agenda placed on clinicians from an external source (ie, the government), rather than a change initiative taken up internally by the clinicians. Among the five groups of ideals presented in Textbox 2, a new ideal is emerging because of the implementation of remote consultations. Previously, it was taken for granted that a patient must visit the clinician in person for consultations. However, with opportunities for remote consultation burgeoning, clinicians are becoming aware that making the consultation easily accessible to patients should also be a desired outcome. However, two different patterns in clinicians’ opinions of how VC implementation is affecting the ideals have been identified: (1) endangering the existing ideals of consultation and (2) creating new ideals. Regarding the first pattern, we identified that the aspects can be divided into two parts: clinical aspects, for example, assessing the symptoms and identifying the diagnoses, and interpersonal aspects, which are more subjective and include human interaction, communication, and the importance of small talk. Clinicians have shown certain reservations about VC, as it can reduce the ideal or expected quality of care if conducted regularly in place of PCs. This is illustrated by the following quote:

The fact that the video calls are brief and to the point may sound very positive, there is a negative aspect to that as well. We are actually, very dependent on knowing on who this person is, what kind of patient do we have in front of us, what is their societal context, who do they live with and what do they work with, how is their lifestyles and how would they present their symptoms, and how any disorder they might have that influences the daily life—so a lot of things around those we need to understand. So, if we have to depend on solely on screen for this kind of knowledge that would be limited and that’s a type of quality loss. And that can be harmful in the long run.

clinician 9, during the first round of interviews

Regarding the interpersonal aspects of the consultation, the clinicians had diverse opinions. Some thought that VC can significantly reduce the quality of these aspects, thus affecting the quality of care:

I actually see a great value in that small talk part, and I feel it is important as a doctor to connect with your patient and it increases their will to use the medication that you prescribe, and it enhances the doctor-patient relation. That’s very important for the patient to trust the doctor and I think that part of consults disappears a bit when we are doing it over the phone or video. I think that’s why a lot of my patients have said that they look forward to coming here.

clinician 11, during the first round of interviews

Others agreed that these aspects are important for the treatment, at least to an extent, but felt that VC does not reduce the quality of these aspects:

I would say, they [small talk] contribute, they are kind of an ice breaker, but everybody there really understands why we are here. They are not really here to chat with me, they are there for the treatment. With VC, I manage to have that much chit chat.

clinician 4, during the second round of interviews

Although these opinions are primarily about whether and how VC can endanger the overall quality of care in the consultation service, another stream of thought focuses on whether and how VC can improve the quality of care. Some clinicians emphasized how VC makes the consultation service easily accessible to patients and their family members by reducing the need to travel to the hospital, thereby minimizing travel-induced stress and tiredness, as illustrated by the following explanation by a clinician:

We do not want the children to miss their school and parents to miss their job a lot. Because then disease becomes a big part of their daily life. So, making the treatment as less intrusive as possible is our goal, which can be attained using video consultation under specific circumstance[s].

clinician 1, during the first round of interviews

Thus, VC opens up the possibility for clinicians to minimize patients’ travel-related challenges, which results in the emergence of a new ideal in the consultation service. These two patterns of ideals—potentially harming the care quality and potentially improving the care quality—create opposing effects on clinicians’ decisions about VC. Those who perceive that the potential loss of care quality outweighs the reduction of travel-induced predicaments are more likely to prefer PC over VC when other aspects, which will be discussed later, remain the same. Similarly, those who perceive that the reduction of travel-induced predicaments outweighs the potential loss in care quality will prefer VC over PC. However, the clinicians did not identify any changes in the outcome of the consultation, positing that it was very early to detect whether VC will change the outcome of the consultation:

It will take time to see how really VC affects the consultation in the long run.

clinician 2, during the second round of interviews

The other aspects can be grouped as managerial ones that include dimensions such as waiting time and facility use. Some clinicians thought that the durations of TC and VC are shorter than that of PC. Thus, according to them, using VC and TC, where appropriate, can reduce a patient’s waiting time:

Think about it. A person enters your room, hangs the overcoat, and settles down on the chair. By that time, she is quite relaxed and up for more like a conversation. So, we open up the conversation with how is the weather, how was the travel to the hospital, and then, we start talking about treatment, health, and so on. We don’t do that on video or telephone consultation. So, they [TC and VC] take a shorter time.

clinician 16, during the first round of interviews

In contrast, some clinicians thought that the duration of the consultation is not dependent on the mode of consultation (ie, the duration does not vary depending on whether it is PC, VC, or TC):

Usually, I plan video consultation for 30 minutes, physical for 30 to 60 minutes. But you know, on a given day, telephone consultation can also take 60 minutes. So you cannot generalize. Sometimes, video consultation can take even longer [than physical]. Either the patient or I can have a technical problem, so it takes more time to connect with the patient and keep the talk going.

clinician 12, during the first round of interviews

Finally, the clinicians thought that VC can affect facility use in the hospital. The consultations conducted over the telephone and video do not need a traditional consultation room equipped for physical examination; thus, they can be conducted in either the clinicians’ private office or smaller rooms that are equipped for telephone and video calls without beds and other clinical apparatus:

In our outpatient clinic, we are at the border of the capacity, so if we are to continue to expand the way we have in the last 10 years with 5–7% the number of patients, it would not work. We have to do something. So to us, the prospect of increasing our activity with telephone and video is a necessity.

clinician 1, during the second round of interviews

However, this outcome cannot be realized until the number of VC and TC reaches a certain level:

But we need a certain volume in order to change the use of a room or to relieve ourselves from hiring rooms from the internal system for that. So, we have not saved anything as of today, it must be in the future.

clinician 15, during the first round of interviews

To summarize, the clinicians shared diverse thoughts on how VC and TC can affect the ideals and outcomes of the consultation. These opinions can be grouped into clinical, interpersonal, and managerial aspects. Depending on a clinician’s perceptions of (1) how different modes of consultation affect each of these aspects and (2) how these aspects affect the overall quality of consultation service, the clinician will make plans for the consultations. In the following section, we have elaborated on how clinicians plan and conduct different consultations.

#### Plans and Actions

Textbox 2 lists 6 different planning criteria that clinicians use to choose the consultation mode. However, not all clinicians consider all these criteria, and their opinions about these criteria are varied. Some clinicians considered patients’ living location as a criterion for choosing VC or TC, whereas others thought this can result in discrimination because patients living closer to the hospital would receive more PC than those living farther away. In this section, we analyzed how the clinicians reasoned for their planning criteria. Here, it is noteworthy that even amid the restrictions of the pandemic, the number of TC was much higher than that of VC, and it continues to be so. Clinicians who were used to TC before VC was introduced often thought that if a physical examination is not required in a consultation, the flexibility and ease that TC offers outweighs the benefit of seeing the patient’s face, as can be seen from the following quote:

We feel that the telephone is sufficient; it works well. Everyone has a telephone, it is easy, everyone knows how to use it, and to start this video consultation, you need to collect email addresses from the patients beforehand, make a call appointment, you have to log on to the tech [the video platform], the patient has to log on to the tech–all that seems like new obstacles without gaining any clinical advantage for them.

clinician 7, during the first round of interviews

In contrast, some clinicians emphasized the importance of seeing patients, thus considering VC to be superior to TC:

To be honest it is very interesting to see patients in their own home, the background. Sometimes I feel like to go to their home and see how they live, if it is tidy or they living in the mess. This is very valuable for the doctors. When you see the patients on video or they come to you, you see a lot of that life that is missed in audio. Most importantly you need to see the face of the patients, this is very important. All that you miss in a telephone consultation.

clinician 5, during the second round of interviews

We identified some changes in the steps of consultation activities (Textbox 2) when a clinician conducts VC or TC instead of PC. The changes in actions were limited to making a video or telephone call instead of taking the patient into the consultation room, communicating with the patient through a device (ie, computer or telephone instead of direct communication), and occasional troubleshooting of technical issues. Although these activities are new to the routine of consultation, they are not unfamiliar to clinicians or something that clinicians need to learn or be trained for. The software used by clinicians to make video calls has been in use at the Pioneer Hospital for long-distance videoconferences for some time. This makes VC easier for clinicians. However, the clinicians sometimes faced technical difficulties in making video calls. An easy work-around for such instances was switching to telephone calls, and the clinicians did not report troubleshooting the issues after the consultation:

As long as I have made the consultation, talked to the patient, I do not go back on thinking why Skype did not work this time.

clinician 4, during the first round of interviews

Besides these changes in some of the steps of consultation activities, we did not identify any changes in terms of the role that clinicians have in the consultation. Textbox 3 provides an overview of how the clinicians navigated through these 4 aspects of the consultation routine in PC, VC, and TC.

**A summary of the routine of consultation incorporating in-person consultation (PC), video consultation (VC), and telephone consultation (TC).**

**Ideals**

- The ideals that clinicians held for consultation can be divided into three aspects: clinical, interpersonal, and managerial, and all the three aspects affect the quality of care.
  Clinical aspects include diagnoses and treatments.
  Interpersonal aspects include human interaction, communication, trust, safety, and comfort.
  Managerial aspects include waiting time and facility use.
- Introduction of VC prompts clinicians to consider (1) a new ideal of improving a patient’s accessibility to the consultation service by reducing travel-induced stresses and time spent for the consultation and (2) whether VC can potentially reduce the quality of human interaction and communication and weaken the patient’s experience regarding safety and level of comfort in consultation.

**Plans**

- Clinicians had mixed opinions on the potential merits and demerits of VC and its position between the two other modes of consultation (ie, TC and PC) that existed before the introduction of VC.
- Totally, 6 different planning criteria have been identified in Textbox 2, and the individual clinicians used a different combination of these criteria when choosing a mode of consultation.

**Actions**

- According to the clinicians, conducting VC does not require rigorous training and does not add to or omit any existing role. The minor changes in the actions in a consultation do not seem to play a significant role in clinicians’ choice of consultation mode.

**Outcomes**

- The clinicians could not identify any changes in the outcome of consultation because of the introduction of VC. Cost reduction was an evident outcome of remote consultation in hospitals. Although clinicians were aware of the importance of cost efficiency to run the hospital, they did not consider this as a desired outcome of consultation service.

## Discussion

### Principal Findings

This study found that clinicians’ choice of consultation mode depends on clinical, interpersonal, and managerial aspects and the changes identified in their daily consultation-related tasks are simple to manage. However, when they were faced with technical difficulties in conducting VC, they preferred to switch to TC instead of spending time in fixing the technical issue. Although the health authority and hospital management have emphasized the cost efficiency of VCs, the clinicians did not consider it to be a deciding factor for consultation mode. Before the introduction of VC in the hospital, the clinicians did not find it necessary to change the ongoing consultation service that included only PC and TC, and they could not identify any change in the outcome of the service after the introduction of VC. We identified that the clinicians neither rejected VC nor embraced it, but rather accepted it with caution and a reluctant attitude. They reasoned their attitude toward VC in various ways that were not entirely consistent. The way the clinicians reasoned their use of different modes of consultation appears to be paradoxical. On the one hand, while choosing PC over VC, they posited that VC could harm the quality of care in the long run. They argued that not being able to meet the patient in person could mean that the communication and interaction between the patient and clinicians become less rich and informative. In contrast, they justified using TC over VC, positing that telephones are easier and more flexible to use, which implies that they did not value the ability to see the person on screen in VC. It seems that the clinicians were caught between the importance of seeing and meeting the patients during the consultation and the ease and flexibility of using a particular mode. Consequently, the clinicians have developed their personal favorites and preferences, which they justified using different reasonings.

### Explanation of Choices Made by Clinicians

In this section, we provide a plausible explanation for the clinicians’ diverse and inconsistent choices regarding consultation modes. When compared with health care technology, such as a minimally invasive cardiac surgery technique [37], the technology for VC requires only simple changes in clinicians’ routines. Studies have shown that technology that causes disruptive changes in routine actions or limits professional autonomy is harder to implement and often ends up being abandoned or used in a limited manner, as opposed to finding large-scale application [10,37,38]. In the case of adopting VC, the clinicians did not feel that their roles or professional autonomy had been altered in any way, and they did not feel the need for formal learning or training to conduct VC. Therefore, VC was not dismissed by clinicians because of disruptive change in routines. Although VC implementation does not provide any strong reason to dismiss it, it does not provide any explicit benefit for the clinicians to adopt it immediately. We cannot ignore the growing number of randomized controlled trial studies that show how VC impacts diagnosis-specific clinical efficacy and safety [39,40]. However, there is an increasing discrepancy between experimental trials and the experience of remote consultation as a regular service [12,41,42]. The clinicians in our study wondered about the long-term effects of VC on the quality of care. Moreover, we identified that it is not only how VC impacts the clinical aspect that needs to be considered but also how it impacts the interpersonal and managerial aspects. Scientific evidence on the long-term effect of VC on all the three aspects is inadequate according to clinicians in our study, and this is consistent with previous studies [41,42].

Another important issue to consider is that the objective of VC implementation in the hospital was primarily economic, a factor that the clinicians did not feel strongly or care about in terms of treating patients. Other interventions, such as computed tomography scanners [43] or minimally invasive surgery techniques [37], have demonstrated clear improvements in the level of clinical care from the beginning of their implementation. Previous studies have shown that when medical professionals realize that an intervention can improve their clinical practices, they are less dismissive of the changes and eager to incorporate the new practice while trying to minimize the changes in their routine [15]. VC does not offer any such explicit incentive to clinicians. Therefore, the clinicians have not embraced this new mode of consultation with much enthusiasm, which explains why the clinicians were not using VC to a great extent before lockdowns were imposed in March 2020.

**Figure 2:**
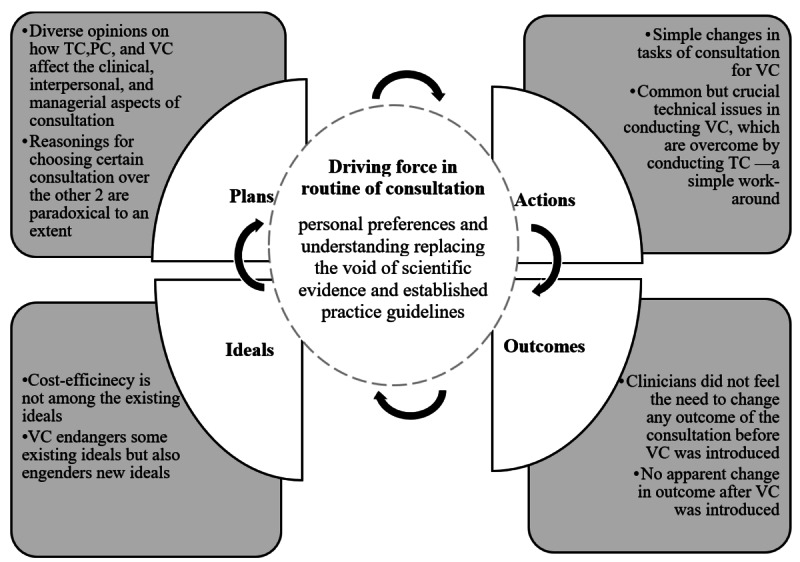
Performative model of routine for services using in-person consultation (PC), telephone consultation (TC), and video consultation (VC).

We posit that the absence of sufficient scientific evidence, clear understanding of the merits and demerits of VC, and standard practices and shared norms for conducting VCs have created a void in the consultation practice. This void leads clinicians to use their personal judgments and preferences to justify their choices regarding PC, VC, and TC. Thus, a wide variety of moderately paradoxical reasons can be identified from the clinicians’ accounts of how they choose the mode of consultation. This void—created by lack of evidence and standard practice and shared norms—is a unique phenomenon for clinical practice. These factors are the pillars of the medical profession, and they drive the medical practitioners’ decision-making [14,44]. In the absence of these pillars, each clinician uses their professional autonomy and agency to interpret the effects of VC and decide how and when to use each consultation method [45]. This explains the variety and paradoxes seen in their reasoning regarding the choice of consultation. Figure 2 shows an updated framework for the routine of consultation (ie, findings in plans, actions, outcomes, and ideals) and the driving force behind the current routine that incorporates all three modes of consultation.

### Comparison With Previous Studies

The literature relevant to VC has been discussed previously; therefore, in this section, we compare this study with previous studies focusing on clinicians. One of these studies [2] examined how clinicians perceive the limitations of VC and how the relationship between clinicians and patients may change when VC replaces PC. The primary finding was the set of disturbances and limitations experienced by clinicians who have experienced VC. The study provided in-depth analysis of the disturbances and limitations of VC and revealed the consequences of the consultation if such disturbances persist. Moreover, the study also identified that the responsibility of creating a suitable ambiance for consultation is shared by both the clinician and patient in VCs, proposing that if clinicians do not consider the patient’s ability to create a suitable environment, the consultation may have reduced quality. A second study conducted by the same group of researchers focused specifically on the selection criteria clinicians used to choose patients for VC [46]. Our findings confirm the selection criteria used by the clinicians in their study when choosing patients. However, our study further generates new insights by examining how clinicians navigate through a consultation service when they have three alternative modes to provide the service.

First, we examined clinicians’ choices, not only regarding VC but also regarding the total service (ie, PC, TC, and VC), showing that the availability of TC along with PC adds paradoxes in clinicians’ choice of consultation. By using a performative model of routine as the theoretical lens, we then identified how clinicians compare the goal of VC with their ideals and expected outcomes of consultations and, consequently, plan and conduct the consultations. Thus, in addition to the barriers and patient selection criteria, our analysis identified other criteria for choosing the consultation mode, including interpersonal and managerial ones and a clinician’s personal preferences and previous experience with TC. Our analysis of an individual clinician’s routine of consultation also reveals the wide variations that exists in clinicians’ sense-making processes regarding the different modes of consultation and their opinions on the potential benefits and harms these modes can cause. Finally, we provide a plausible explanation for the varied and moderately paradoxical opinions of clinicians by using the literature on the medical profession and professional organizations.

### Strengths and Limitations of the Study

This study contributes to the eHealth literature by generating deeper insights into clinicians’ decision-making processes regarding remote and PC, which has significant effect on the sustainability of the large-scale implementation of remote consultations. Once the variety in clinicians’ opinions about the different consultations can be minimized, the uncertainty of how and when to use each mode of consultation can be reduced, making it more likely that all modes of consultation will become routine (ie, the flow of actions without a less active comparison between outcomes and ideals and adjustments in plans) [47], hence, making it become sustainable. We argue that to minimize the variety in opinion, clinicians require the scientific, long-term evidence on the effect of VC and TC not only on clinical but also on interpersonal and managerial aspects of the consultation, which have not been in focus in the literature.

Moreover, our findings are useful for health care IT implementation in general. To advance IT implementation practice and research, it is essential to identify the theoretical mechanisms and contingencies of IT implementation [27]. This is not addressed by most of the current health care IT literature [48,49]. We provide an explanation for the low number of large-scale IT adoption and sustainable implementation projects in health care organizations: when the objective of an IT implementation program is not directly aligned with the ideals that clinicians hold for a certain health care service, clinicians do not immediately welcome the implementation, even if the IT does not threaten their professional autonomy or complicate their existing routines. Instead, they seek reasons to dismiss or adopt it. In these situations, if enough evidence or uniform understanding of the benefit and harm caused by the IT is nonexistent, the professionals can rely on their individual judgment and personal preferences to decide how and to what extent they adopt the IT. Consequently, diverse opinions emerge, including some paradoxical ones, resulting in an uncertain future for sustainable large-scale implementation.

The limitation of this study is that it focuses on a single health care organization. Although the chosen organization is one of the largest and most prominent hospitals in Norway, one can question the extent to which our findings and explanations are valid for other hospitals worldwide. To minimize this limitation and enhance the usability of the study, we provided a detailed description [50] of the national and local contexts of the hospital. We aimed to provide readers with good understanding of the context and demonstrate that the findings and explanations are embedded within the context. Thus, the findings of this study can be compared and contrasted with those of future studies from similar or different contexts.

### Directions for Future Studies

We posit that it is crucial to investigate and identify the efficacy of remote consultations in their entirety so that the potential benefits can be realized and exploited to the maximum and the potential harms can be minimized. Our findings emphasize the need for future studies on VC in several directions: (1) the long-term clinical effect of remote consultation (eg, VC and TC), (2) the effect on the interpersonal aspects of consultations and how these aspects affect the quality of care in consultation, and (3) the effect on managerial aspects and how remote consultation can improve the management and organization of consultation services. Studies conducted in these directions can help provide scientific evidence for a different mode of consultation and a strong base to generate, share, and help to develop the values and norms about how clinicians practice consultations using multiple modes.

Our study also reveals that besides conducting studies in these areas, a strong focus is needed on how to disseminate these findings among clinicians. If clinicians are not aware of the scientific evidence, their process of choosing the consultation mode will remain the same. On the one hand, a separate stream of research on how to disseminate scientific evidence and good practices for different consultation modes would be beneficial. In contrast, as a crucial step in implementing VC, the management of the hospital needs to consider facilitating learning, sharing of experiences (good or bad), and dissemination of research.

### Conclusions

The research and practitioner communities worldwide are deeply engaged in anticipating how VC will be adopted in hospitals as a regular service and how it will change the consultation service. This study contributes to this ongoing conversation by including new insights into how, on a daily basis, clinicians make sense of the availability of the three modes of consultation (ie, PC, TC, and VC) and how they reason their choice of a mode over others. We conclude that as a digital intervention, VC does not drastically change the routine of consultation for clinicians. However, it also does not provide an immediate clinical benefit. Thus, clinicians neither dismiss the option of VC nor feel an urgency to adopt it. The study also revealed the absence of sufficient scientific evidence on the long-term merits and demerits of VC, standard practice, and shared norms regarding when to use (and not to use) VC. Under this circumstance, clinicians tend to rely on their personal assessment and preferences to decide the mode of consultation, which leads to wide variety in clinicians’ choice of consultation mode. This variety risks the quality of the consultation service and patient satisfaction because patients with similar diagnoses may receive different forms of health care, depending on the clinicians they are consulting. Therefore, this study calls for future studies on the long-term effect of VC, not only regarding clinical attributes but also interpersonal and managerial attributes. We also emphasize that the dissemination of these studies among clinicians is equally important because these results can answer the questions they ask about the long-term effect of VCs, and consequently, develop best practices and share the norms for this digital service.

## Appendix

Multimedia Appendix 1 Interview guide consisting of three sets of questions for clinicians, patients, and advisers.

## Abbreviations

IT: information technology
NSD: Norwegian Center for Research Data
PC: in-person consultation
REK: Regional Committee for Medical and Health Research Ethics
TC: telephone consultation
VC: video consultation
