# Supplementary material for: Individuals’ Perceptions as a Substitute for Guidelines and Evidence: Interview Study Among Clinicians on How They Choose Between In-Person and Remote Consultation
Source: JMIR Form Res. 2022 May 25;6(5):e35950. doi: 10.2196/35950 (PMC9178453; doi:10.2196/35950)
Supplement: Multimedia Appendix 1 [file formative_v6i5e35950_app1.docx]

# Multimedia Appendix 1. Interview guide consisting of three sets of questions for clinicians, patients, and advisers.

**Background Information**

1. Current position in the polyclinic
2. Duration of employment
3. Job responsibilities
4. Connection between your income and, number and type of consultation.
5. Connection between hospital’s income and, number and type of consultation.

**Questions on work process**

1. Can you describe a usual work-day for you?
2. If we go into more detail now, what are the specific tasks you do in relation to a consultation
   1. Before you meet the patient for the consultation
   2. During the consultation
   3. After the patient leaves
3. Duration of these meetings and whether documentation/recording are included in this duration (in principle and practice).
4. Can we talk about the facilities/physical capacities of polyclinic? (Room arrangement during the consultation and other times.)
5. Can you recall when you first heard about video consultation here in the hospital?
6. Were you used to doing telephone consultation before the idea of video consultation came?
7. Why do you think that video consultation is important for the hospital?
8. Can you identify changes in your tasks when you meet patients over the telephone and through video call, (e.g. duration of consultation, documentation)?
9. How is it decided whether the next consultation for a patient would be physical, video or telephone?
10. What are the things (criteria) that you consider for this decision?
11. Is there any guideline from the management regarding the use of video consultation and telephone?

**Questions on the perception of different consultations**

1. Can you share your experience with video calls?
2. Can you compare your experience among in-person, telephone, and video consultation?
3. Do you think there is any benefit of video calls over telephone calls and vice versa? (If yes, what are those).
4. Do you think there can be some risk associated with video calls and telephone calls? (If yes, what are those).
5. As the number of video calls got increased due to Covid, do you think it will go down once/when we overcome the virus?
6. Do you think the conditions for telephone and video consultation can be standardized in the future, e.g. based on the type of diagnosis, patient’s geographical location.
7. Do you think video consultation and/or telephone consultation can contribute to improving the capacity and waiting list issue in the policlinic and if yes, how?

# Interview Guide for Advisers (not in direct communication with patients)

**Background**

1. What position do you currently hold?
2. How long have you worked at the hospital?
3. What are your job responsibilities?

**Hospital management in general**

1. What are the challenges in the hospital (at present and in near future)?
2. How does the management see digital services about these challenges?
3. What are the ongoing digitalization projects in the hospital at this moment?

**Video Consultation**

1. If we take video consultation in particular, how does the implementation of this service benefit the hospital, (as you are trying to implement it at present)?
2. Can you share about the implementation process of video consultation? (find out: when it started, who brought the idea, who are involved in planning, who are involved in execution)
3. To what extent the clinicians are participating in the implementation?
4. Are there any challenges in implementing the video consultation?
5. Why is this implementation so important for you (the hospital)?
6. Can you reflect on how have Covid-19 Pandemic affected the implementation of video consultation?
7. Does the management provide any standard guideline on the use of in-person, telephone, and video consultation?
8. Why do you think that the number of telephone consultations is higher than that of video consultations?
9. What happens when the hospital cannot reach the annual goal for video consultation? (find out the consequences, the communication between management and the clinicians)
10. What are the future plans for video consultation? (how will it be continued; how will they keep the momentum gained by the Covid-19 pandemic)

# Interview guide: Patients

**Background**

1. Is it okay for you to briefly mention your diagnosis for which you receive consultation service from the hospital? (if not, go to the next question)
2. How long has the treatment been going?
3. How often do you have a consultation service?
4. Do you visit the same clinician for all consultations?

**Video consultation**

1. Have you ever been in a telephone consultation and/or a video consultation?
2. Can we talk about your experience with the consultations that are not in-person visits?
3. Have you experienced any difference between in-person and video consultation?
4. Have you experienced any difference between in-person and telephone consultations?
5. Have you experienced any difference between telephone and video consultation?
6. Which type of consultation do you prefer most and why?
7. Which type of consultation do you prefer least and why?
8. How is it decided whether the next consultation will be a telephone/video/in-person consultation?
9. How involved are you in this decision-making process?
10. Do you miss anything in the current consultation service?
11. Do you have any suggestions to improve the service?
